# Research Progress on Coronary Artery Injury and Myocardial Ischemia in Multisystem Inflammatory Syndrome in Children

**DOI:** 10.3390/cimb48060558

**Published:** 2026-05-26

**Authors:** Jirong Liu, Nanyan Mao, Yaru Cui, Yiyao Bao, Chao Tang

**Affiliations:** National Clinical Research Center for Children and Adolescents’ Health and Diseases, Children’s Hospital, Zhejiang University School of Medicine, Hangzhou 310052, China; 6514166@zju.edu.cn (J.L.); 6511125@zju.edu.cn (N.M.); 6514118@zju.edu.cn (Y.C.)

**Keywords:** multisystem inflammatory syndrome in children (MIS-C), coronary artery injury, coronary artery aneurysm, myocardial ischemia, inflammatory storm

## Abstract

Multisystem inflammatory syndrome in children (MIS-C) is a severe systemic inflammatory complication triggered by prior SARS-CoV-2 infection. It predominantly affects the cardiovascular system, and coronary artery injury, myocardial dysfunction, and myocardial ischemia are closely associated with disease severity and clinical outcomes. This article reviews the immunopathological characteristics and clinical manifestations of MIS-C-related coronary artery lesions, including coronary artery dilation and aneurysm formation, as well as the key pathophysiological mechanisms leading to myocardial ischemia. Based on recent clinical and translational research, we summarize current approaches to diagnosis, risk stratification, acute medical management, and long-term follow-up strategies. By synthesizing updated evidence, this review aims to provide theoretical support and practical clinical guidance for the early identification, timely intervention, and optimized management of affected children, ultimately improving their long-term cardiovascular prognosis.

## 1. Introduction

Multisystem inflammatory syndrome in children (MIS-C) is a novel hyperinflammatory condition that emerged during the COVID-19 pandemic, characterized by persistent fever, multisystem organ involvement, and markedly elevated inflammatory markers, temporally associated with prior SARS-CoV-2 infection [1]. The pathogenesis of MIS-C is uniquely linked to SARS-CoV-2, as the viral spike protein exhibits superantigen-like properties that trigger broad non-specific T-cell activation, distinguishing it from other coronavirus infections. While acute COVID-19 infection is mild, MIS-C is a rare but severe condition that can arise several weeks after infection and presents with several inflammatory symptoms. The diagnostic criteria for MIS-C are age under 21 years, persistent fever, evidence of significant inflammation, involvement of at least two organ systems, no other plausible explanation, and a recent SARS-CoV-2 infection [1]. The clinical symptoms are skin and mucosal symptoms (rash and conjunctival congestion), digestive symptoms (abdominal pain, vomiting and diarrhea), neurological symptoms and cardiovascular symptoms. Most patients present with fever (99.4%), followed by gastrointestinal symptoms (85.6%), and cardiovascular involvement (up to 80%) [2]. It was thought children were resistant to severe COVID-19, but MIS-C suggests they may also be at risk for systemic inflammation.

Cardiovascular involvement is a common and severe manifestation of MIS-C. Cardiovascular disease, cardiac insufficiency and shock occur often [3,4]. Up to 80% of MIS-C cases are associated with myocardial injury (elevated cardiac troponin and B-type natriuretic peptide levels), pericarditis, arrhythmias, and coronary artery involvement, often requiring vasopressor support [3]. Cardiovascular involvement is reported to range from 4 to 27.78%, often as dilation and aneurysm [5,6]. Cardiovascular involvement is more common in the acute stage of MIS-C than in the classic Kawasaki disease. Cardiovascular injury and dysfunction are more frequent in the acute stage of MIS-C [5]. Cardiovascular issues like myocardial ischemia and cardiac insufficiency are leading causes of intensive care admission and death in children [7]. More than half of patients (56.3%) develop shock and require vasopressor support [2]. In Mexican children, almost half (45.6%) required admission to a pediatric intensive care unit [8].

While MIS-C shares certain pathological features with Kawasaki disease, several key differences have been identified, including a more intense cytokine storm, distinct T-cell activation profiles, and a higher prevalence of myocardial dysfunction in MIS-C. Both conditions are thought to be caused by immune dysregulation and cytokine storms, but the immune activation may be more extensive and intense [1,9]. Research suggests that the cytokine profile of children with MIS-C is higher than that of severe COVID-19 in adults and that it has increased markers of recent T-cell activation (soluble interleukin-2 receptor), although the humoral immune response to SARS-CoV-2 is typically strong. Genetic factors may also contribute to MIS-C susceptibility; while certain genetic variants are shared between MIS-C and Kawasaki disease, distinct genetic predispositions specific to MIS-C have also been identified. Shock may occur earlier, and the risk of myocardial damage (high-sensitivity troponin T) and decreased left ventricular ejection fraction may occur less frequently [10,11]. Understanding mechanisms, clinical features and outcomes of coronary artery injury and myocardial ischemia related to MIS-C is crucial for designing effective treatment plans. We review current research on coronary artery injury and myocardial ischemia, including epidemiology, clinical features, assessment of coronary artery lesions, management, long-term prognosis and follow-up.

## 2. Epidemiological, Clinical Characteristics and Overview of Cardiovascular Involvement of MIS-C

### 2.1. Epidemiological Features and Diagnostic Criteria

MIS-C is rare but severe hyperinflammatory syndrome associated with SARS-CoV-2 infection that affects school-age children and adolescents [2]. The median age of MIS-C patients is approximately 8 years, with a slight male predominance (approximately 58.9% of cases) [2]. The syndrome usually manifests 2–6 weeks after SARS-CoV-2 infection, which correlates to peak community infections [12]. The WHO and CDC have established diagnostic criteria including persistent fever, increased inflammatory markers like C-reactive protein, and clinical evidence of involvement in at least two organs [13,14]. Cardiovascular involvement is common, affecting up to 80 percent of MIS-C patients (myocardial dysfunction, pericardial effusion, valve regurgitation, and coronary artery abnormalities are key diagnostic features) [15].

### 2.2. Clinical Manifestations of the Cardiovascular System in MIS-C Acute Phase

Clinical manifestations of the cardiovascular system in the acute phase of MIS-C range from mild to severe and encompass shock (cardiogenic and distributive), significant decrease in left ventricular systolic function (myocardial depression), arrhythmias and increase in serum markers of myocardial injury such as troponin and BNP/NT-proBNP [16]. A German multicenter study showed that patients could be classified according to clinical manifestations into shock-like, Kawasaki-disease-like and non-complex high-inflammation types. The shock-like patients had the highest BNP levels, and Kawasaki-disease-like patients had the largest increase of troponin [16]. A significant decrease in left ventricular function is the main feature of myocardial depression in the acute phase, and up to 56.32% of patients in the acute phase had myocardial dysfunction [17]. This dysfunction is often associated with elevated serum markers of myocardial injury (especially troponin and BNP/NT-proBNP), and abnormal elevation of these markers is one of the key laboratory characteristics distinguishing MIS-C from typical Kawasaki disease [18,19]. Conduction abnormalities, including atrioventricular block, are also common and more likely to occur in some children who need to be admitted to the intensive care unit and have ventricular dysfunction [20]. Coronary artery edema and dilation are frequently observed during the acute inflammatory phase. Systematic review summary data showed that 17.83% of MIS-C children had coronary artery dilation and 6.85% reached coronary artery aneurysms [17]. These abnormalities are the basis for subsequent myocardial ischemia and long-term cardiovascular risk [4]. These abnormalities play a role in coronary artery disease [4]. The severity of clinical manifestations varies, ranging from only presenting with mild myocarditis to severe cardiogenic shock requiring vasoactive drugs and mechanical circulatory support (such as extracorporeal membrane oxygenation) [21]. It is worth noting that, compared with Kawasaki disease shock syndrome, shock tends to occur earlier in the disease course in children with MIS-C compared to Kawasaki disease shock syndrome, and the incidence of myocardial damage and decreased left ventricular ejection fraction is higher [22,23]. Despite the critical condition, with timely identification and treatment, the short-term prognosis of most children is good and the mortality rate is low [2,24].

Mucocutaneous manifestations, including rash, conjunctival injection, oral mucosal changes, and extremity edema or erythema, are frequently observed in MIS-C and overlap significantly with the clinical presentation of Kawasaki disease. A study by Rao et al. compared disease severity between MIS-C patients with and without mucocutaneous findings and demonstrated that the presence of these manifestations may carry prognostic significance [25]. Specifically, patients presenting with prominent mucocutaneous features tended to have a distinct clinical course, potentially reflecting differences in the underlying immunopathological mechanisms [25]. Recognizing these dermatological findings as part of the MIS-C spectrum not only aids in early clinical recognition but may also contribute to refining risk stratification. Further prospective studies are warranted to elucidate whether specific patterns of mucocutaneous involvement can reliably predict cardiovascular outcomes or guide therapeutic decisions in MIS-C patients [25].

## 3. Pathophysiological Mechanisms of Coronary Artery Injury and Myocardial Ischemia Related to MIS-C

### 3.1. Immune Dysregulation and “Cytokine Storm”

The primary driver of MIS-C is abnormal immune response after SARS-CoV-2 infection that activates CD4+ and CD8+ T cells, Th1/Th17 polarization and excessive monocyte/macrophage activation [26]. It induces high levels of proinflammatory cytokines, i.e., IL-1β, IL-6, IL-10, TNF-α and IFN-γ [27]. The systemic inflammation damages the vascular endothelial and myocardial cells and increases the vascular permeability, vasculitis and microvascular dysfunction. In the children affected the serum levels of some proinflammatory cytokines lead to coronary artery injury and impaired myocardial perfusion [10]. Together, these processes contribute to MIS-C-associated myocardial dysfunction and coronary artery involvement [28]. MIS-C is similar to Kawasaki disease in terms of high cytokinesis, but its cytokine profile may be more subtle, suggesting that there is a distinct immune activation pathway [27].

In addition to cytokine-driven inflammation, oxidative stress plays a significant role in the pathogenesis of MIS-C-related cardiovascular injury. SARS-CoV-2 infection triggers the excessive production of reactive oxygen species (ROS) and reactive nitrogen species (RNS), which overwhelm endogenous antioxidant defense systems and contribute to endothelial dysfunction, lipid peroxidation, and myocardial cell damage [29]. Elevated levels of oxidative stress markers, including malondialdehyde (MDA) and protein carbonyls, have been observed in MIS-C patients, along with reduced antioxidant capacity, as indicated by decreased glutathione (GSH) levels and superoxide dismutase (SOD) activity [29]. The resulting oxidative damage exacerbates the inflammatory cascade, creating a vicious cycle that amplifies vascular endothelial injury and promotes coronary artery damage. These findings suggest that antioxidant strategies, such as N-acetylcysteine or vitamin C supplementation, may represent a potential adjunctive therapeutic approach to mitigate oxidative injury in MIS-C, although further clinical studies are needed to validate their efficacy [29].

At the molecular level, the SARS-CoV-2 spike protein contains a high-affinity superantigen-like motif within its S1 subunit that directly binds to the T-cell receptor (TCR) beta chain variable region (TRBV), particularly TRBV11–2, resulting in polyclonal T-cell activation that bypasses conventional antigen-specific recognition [26]. This superantigen-mediated activation triggers downstream signaling cascades, including the nuclear factor-kappa B (NF-kappaB) and Janus kinase–signal transducer and activator of transcription (JAK-STAT) pathways, leading to the massive transcriptional upregulation of proinflammatory cytokines, particularly IL-6, TNF-alpha, and IL-1beta [10]. Concurrently, the mitogen-activated protein kinase (MAPK) pathway is activated, further amplifying the inflammatory response. The spike protein also engages Toll-like receptor 4 (TLR4) on monocytes and macrophages, triggering MyD88-dependent signaling that contributes to the hyperinflammatory state characteristic of MIS-C. These molecular cascades collectively drive the “cytokine storm” that underlies the cardiovascular pathology observed in affected children [27].

### 3.2. Vascular Endothelial Injury and Hypercoagulable State

Viral antigens, such as spike protein or autoantibodies, can attack the vascular endothelium by molecular mimicking [30]. Activated endothelium cells with high adhesion molecules and procoagulants are responsible for white blood cell infiltration, local thrombosis, coronary artery inflammation and stenosis [31]. Children with MIS-C often exhibit high D-dimer levels, thrombocytopenia or functional abnormalities, and clear hypercoagulable state increasing coronary artery thrombosis [32]. Endothelial injury and hypercoagulable state contribute to coronary artery thrombosis for acute myocardial infarction [32]. Autoantibodies against cardiovascular, gastrointestinal and endothelial antigens are also considered critical in accelerating inflammation and damage [10]. Clinical studies show the coronary artery dilation rate is 15.2% in MIS-C children, with structural changes contributing to thrombosis [33].

At the molecular level, SARS-CoV-2 spike protein binds to angiotensin-converting enzyme 2 (ACE2) on endothelial cells, leading to ACE2 downregulation and subsequent dysregulation of the renin–angiotensin–aldosterone system (RAAS). This results in elevated angiotensin II levels, which promotes vasoconstriction, oxidative stress, and endothelial activation via the AT1 receptor [31]. Activated endothelial cells upregulate adhesion molecules, including intercellular adhesion molecule-1 (ICAM-1), vascular cell adhesion molecule-1 (VCAM-1), and E-selectin, which facilitate leukocyte adhesion and transmigration into the vessel wall [30]. Furthermore, the inflammatory milieu induces endothelial expression of tissue factor (TF), triggering the extrinsic coagulation cascade, while simultaneously reducing the expression of thrombomodulin and tissue factor pathway inhibitor (TFPI), shifting the endothelium toward a prothrombotic phenotype [32]. The release of ultra-large von Willebrand factor (vWF) multimers from activated endothelial cells further promotes platelet adhesion and microthrombus formation. Additionally, autoantibodies targeting endothelial cell antigens, including antiendothelial cell antibodies (AECAs) and anticardiolipin antibodies, have been detected in MIS-C patients, suggesting that molecular mimicry between SARS-CoV-2 proteins and host endothelial antigens may contribute to the vasculitic process [10], as shown in Table 1.

### 3.3. Similarities and Differences in the Pathological Mechanisms of Kawasaki Disease and MIS-C

**Figure 1:**
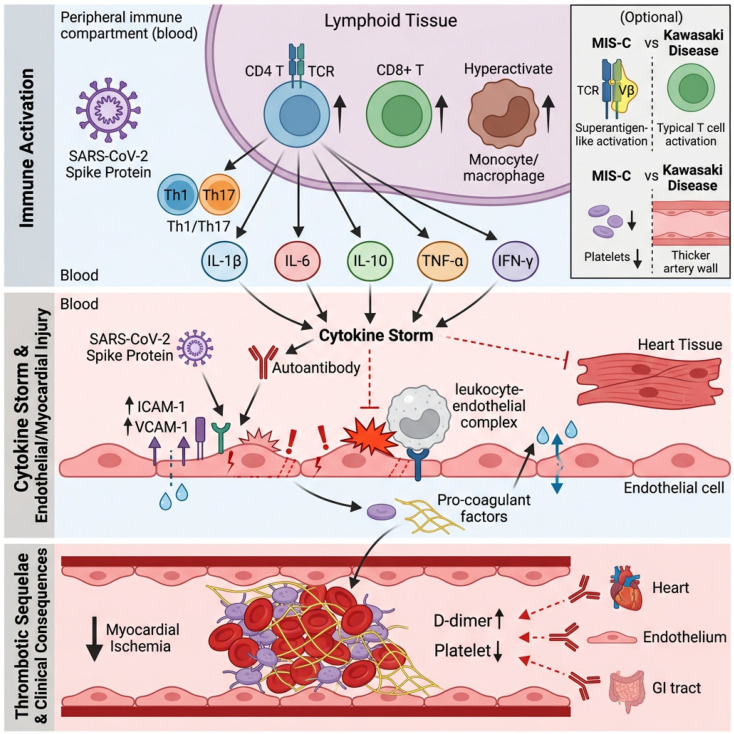
Pathophysiological mechanisms of coronary artery injury and myocardial ischemia related to MIS-C. This figure illustrates the key immunopathological and pathophysiological processes underlying coronary artery damage (including dilatation and aneurysm formation) and subsequent myocardial ischemia in children with multisystem inflammatory syndrome (MIS-C) following SARS-CoV-2 infection. (↑ increase ↓ decrease).

Both MIS-C and Kawasaki disease involve systemic vasculitis but differ in T-cell activation and cytokine profile. Despite these similarities, the two conditions exhibit distinct clinical features; MIS-C is characterized by higher levels of IL-17A and is more frequently associated with gastrointestinal symptoms, shock, and myocardial dysfunction [27]. Also, the structure and progression of coronary artery aneurysms in MIS-C (typically affecting center of right coronary artery) may differ from Kawasaki disease [34]. Superantigen-like response to SARS-CoV-2 is considered as a novel potential trigger for MIS-C, possibly leading to strong non-specific T-cell activation through a biased T-cell receptor V region [10,26]. Kawasaki disease targets only coronary arteries, and myocardial dysfunction is more common in MIS-C. Coronary artery sequelae in MIS-C usually resolve within a few weeks, and coronary artery lesions in Kawasaki disease may last many years [26]. Platelet count is typically lower in MIS-C than in Kawasaki disease, suggesting different immune processes [35], as shown in Figure 1.

## 4. Evaluation, Grading and Imaging Monitoring of Coronary Artery Lesions

### 4.1. Core Diagnostic Value of Transthoracic Echocardiography

Transthoracic echocardiography is the most commonly used imaging method for diagnosing coronary artery lesions in MIS-C [36]. It is important during the acute phase and follow-up of MIS-C, as it enables serial assessment of coronary artery diameter, wall morphology, and aneurysm formation [36]. A systematic review of MIS-C patients showed that up to 28% of children had coronary artery dilation (Z value 2) at admission [37]. Quantitative coronary artery dilation with a corrected Z value based on body surface area is necessary, according to Kawasaki disease management guidelines (Z value 2.5 means dilation, 5.0 means small aneurysm, 10.0 means large aneurysm) [38]. One hundred and fifty-seven children with symptomatic or mild COVID-19 had dilation (Z value > 2.0), indicating the importance of coronary artery assessment even in non-MIS-C infected children [39]. Echocardiography must also assess ventricular function, pericardial effusion, and valve condition, as MIS-C often has significant myocardial dysfunction. A meta-analysis indicated that 55.3% of children had myocardial dysfunction [40], as shown in Table 2.

### 4.2. Application of Advanced Imaging Techniques

For children with MIS-C who have uncertain echocardiographic results or complex coronary artery lesions, cardiac magnetic resonance imaging (CMR) provides a more precise view of proximal coronary artery anatomy and non-invasively evaluates myocardial edema, fibrosis and perfusion [36]. CMR is particularly helpful for subclinical myocardial injury as a systematic review showed that CMR studies detected myocardial edema and/or fibrosis up to 12 months post MIS-C [41]. Coronary CT angiography (CCTA) is useful for mid-lower segment coronary artery lesions and calcification, although radiation exposure may be considered [36]. These advanced imaging techniques are reserved for specific clinical issues rather than routine screening. Clinical decisions still depend mostly on transthoracic echocardiography [36]. A mid-term cardiovascular outcome comparison study between MIS-C and Kawasaki disease showed that advanced imaging can help to assess subclinical myocardial injury [42].

### 4.3. Dynamic Monitoring Strategy and Risk Stratification

**Figure 2:**
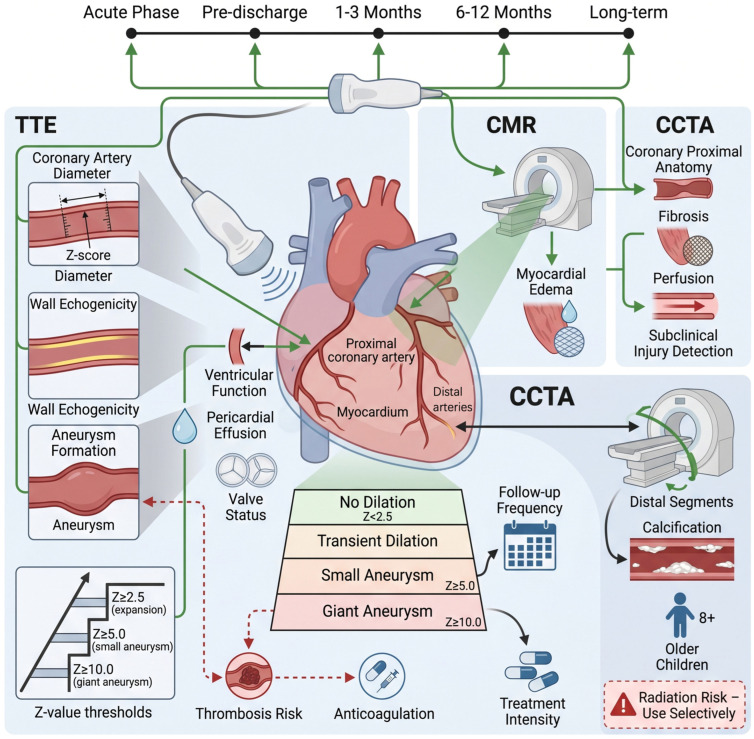
Evaluation, Grading and Imaging Monitoring of Coronary Artery Lesions in Children with MIS-C. This figure summarizes the assessment criteria, grading standards and imaging surveillance strategies for coronary artery abnormalities in patients with multisystem inflammatory syndrome in children (MIS-C).

Since MIS-C coronary artery lesions change rapidly, routine monitoring is needed. Echocardiographic follow-up should be scheduled at acute diagnosis (before discharge), 3 months after discharge, 6–12 months after discharge and after any change in inner diameter [43]. Many cardiac disorders such as coronary artery lesions resolve within 6–9 months after discharge, while some persist as coronary artery abnormalities at 18–24-month follow-up [41]. Treatment and follow-up frequency are usually determined by the maximum Z value of coronary artery lesions (e.g., no dilation, transient dilation, small aneurysms, large aneurysms) [38]. This stratification determines intensity of antiplatelet or anticoagulant therapy and follow-up frequency. Children with coronary artery aneurysms need to monitor risk of thrombus formation and anticoagulation management [44]. In a multicenter prospective cohort study, coronary artery involvement in MIS-C was generally transient, but long-term monitoring based on risk stratification is still needed [42], as shown in Figure 2.

## 5. Manifestations, Diagnosis and Acute Management of Myocardial Ischemia

### 5.1. Biochemical Markers for Myocardial Ischemia and Injury in MIS-C

A meta-analysis by Zhao et al. demonstrated that elevated cardiac markers, particularly troponin and BNP/NT-proBNP, serve not only as diagnostic indicators but also as valuable prognostic tools in MIS-C [45]. The study found that markedly elevated troponin levels (>100 ng/L) and NT-proBNP levels (>1000 pg/mL) were strongly associated with adverse outcomes, including the need for intensive care admission, mechanical ventilation, and vasopressor support [45]. Serial monitoring of these biomarkers during the acute phase allows for dynamic risk assessment, with persistently elevated or rising levels indicating a higher likelihood of clinical deterioration. Conversely, a rapid decline in biomarker levels following immunomodulatory therapy correlates with favorable clinical response and reduced risk of long-term cardiac sequelae [45]. Integration of cardiac biomarker trends with clinical parameters thus enhances the precision of risk stratification and guides the intensity of monitoring and therapeutic intervention in MIS-C patients.

Serum cardiac troponin (cTnI or cTnT) is a highly sensitive biomarker of myocardial cell injury in MIS-C. During the acute phase of MIS-C, the troponin levels rise rapidly, peaking with disease severity [46]. In Italy, patients with MIS-C characteristics (KCG group) were more prone to myocarditis than typical Kawasaki disease patients, and the troponin-T levels were elevated [46]. A systematic review and meta-analysis found that the prevalence of elevated troponin in MIS-C patients was 76.34%, directly indicating myocardial injury [17]. B-type natriuretic peptide (BNP or NT-proBNP), which reflects ventricular wall stress and volume load, is also frequently elevated in MIS-C patients and may suggest myocardial dysfunction or heart failure, although elevated levels can also result from renal impairment, systemic inflammation, and other non-cardiac conditions [47]. For example, in critically ill children, serum levels of B-type natriuretic peptide and troponin decreased after one week of active therapy (including biological agents), suggesting improvement in myocardial injury and heart failure [48]. An observational cohort study in Oxford, UK found that MIS-C children receiving vasoactive drug treatment had significantly higher peak NT-proBNP (median 11,363 ng/L) than those without vasoactive drug treatment (3741 ng/L), correlating with disease severity and hemodynamic instability [12]. Dynamic monitoring of these cardiac biomarkers is useful for measuring response, identifying high-risk children and predicting cardiogenic shock [4], as shown in Table 3.

### 5.2. Clinical Manifestations and Functional Assessment of Myocardial Ischemia in MIS-C

The symptoms of myocardial ischemia in MIS-C range from mild to severe: persistent fevers, nausea, vomiting, diarrhea, chest pain, arrhythmias and, in severe cases, rapidly dying cardiogenic shock with vasoactive drugs [4,49]. Electrocardiography may show ST-T changes or conduction abnormalities, but these are non-specific and cannot be used alone [4]. Echocardiography measures cardiac structure and function and detects left ventricle systolic dysfunction, valve regurgitation and coronary artery abnormalities [11]. CMR is advantageous because the non-invasive nature and tissue resolution allow it to quantify myocardial involvement. It is the gold standard for diagnosing myocarditis and distinguishing ischemic post-myocardial depression from fibrosis [50]. Heart involvement in MIS-C is often more severe than in typical Kawasaki disease; however, existing data show a good prognosis with cardiac dysfunction often improving in a short period [50].

### 5.3. Comprehensive Treatment Strategy for the Acute Phase

The aim of acute MIS-C treatment is to decrease excessive systemic inflammation and treat vascular and myocardial damage. The first line of treatment typically involves IVIG and glucocorticoids such as methylprednisolone [14]. Most patients improve clinical symptoms and inflammation quickly with IVIG [17]. A British consensus recommendation emphasizes the importance of multidisciplinary team decision making and recommends IVIG and methylprednisolone as first immunomodulatory treatments [51]. Children who are resistant to IVIG or present with severe shock require additional adjuvant immunomodulatory treatment. Biological agents such as IL-1 receptor antagonists (anakinra) and IL-6 receptor antagonists (tocilizumab) have been used for refractory cases and have shown good clinical effects [51,52]. Positive inotropic drugs are indicated for myocardial dysfunction and shock [53]. Because high hypercoagulability and risk of coronary artery thrombosis are associated with acute MIS-C, low-dose aspirin (3–5 mg/kg/day, up to a maximum of 100 mg/day) is recommended for antiplatelet therapy [40]. Children with large coronary artery aneurysms require anticoagulants [54]. Combining immunomodulation, cardiovascular support, and antithrombotic therapy aims at controlling acute inflammation and improving heart function and long-term survival.

## 6. Long-Term Cardiovascular Prognosis, Follow-Up and Management Challenges

### 6.1. Outcome and Long-Term Risks of Coronary Artery Lesions

Most coronary artery dilations in MIS-C cases return to normal within weeks to months after the acute phase [2]. Of 50 MIS-C children, eight developed coronary artery aneurysms (Z value 2.5) during the acute phase and four coronary artery dilations (Z value 2.5) during the acute phase. All coronary artery dilations in these children resolved completely within 8 weeks to 6 months of follow-up [55]. A prospective study from India showed a significant reduction in coronary artery dilations in MIS-C children, from 38.9% during the acute phase to 0.9% at 1-year follow-up [56]. A follow-up study of 127 MIS-C children in Germany and Austria found that most dilations during the acute phase resolved within follow-up, with only a few cases (three) persisting [57]. Children with large coronary artery aneurysms may have long-term risks, as they may cause coronary artery stenosis, thrombosis, myocardial ischemia or sudden death which need life-long anticoagulation and close monitoring [54]. Even if coronary artery morphology returns to normal, severe systemic inflammatory response during the acute phase may have caused long-term damage to vascular endothelial function, and early atherosclerosis should be confirmed by long-term cohort studies [58]. A systematic review indicates that most cardiac abnormalities resolve in 6 to 9 months, and cardiac magnetic resonance imaging studies find persistent myocardial edema and/or fibrosis for up to 12 months, with approximately 10–20% of patients exhibiting persistent subclinical vascular or myocardial injury on advanced imaging [41].

### 6.2. Cardiac Function Recovery and Exercise Endurance

Vaccination against SARS-CoV-2 represents an important consideration in the long-term management of children with a history of MIS-C. Le Marchand et al. analyzed MIS-C cases by vaccination status and demonstrated that COVID-19 vaccination is associated with a reduced risk of developing MIS-C following SARS-CoV-2 infection [59]. While the overall incidence of MIS-C has declined substantially in the post-vaccination era, rare cases of MIS-C have been reported after vaccination, though at significantly lower rates than following natural infection [59]. Current evidence supports the recommendation that eligible children, including those with a prior history of MIS-C, should receive COVID-19 vaccination according to age-appropriate schedules, as the protective benefits against severe SARS-CoV-2 infection and MIS-C recurrence outweigh the minimal risks [59]. However, the optimal timing of vaccination following an MIS-C episode remains an area requiring further study, and decisions should be individualized based on the patient’s recovery status and immunomodulatory treatment history [59].

Accumulating evidence indicates that MIS-C survivors may be at increased risk for developing hypertension during the post-acute phase. Lehman et al. reported that a notable proportion of children developed elevated blood pressure or frank hypertension following MIS-C, which may persist for months after the acute illness [60]. The underlying mechanisms are thought to involve residual endothelial dysfunction, altered vascular reactivity, and ongoing subclinical inflammation affecting the renal and systemic vasculature [60]. Therefore, regular blood pressure monitoring should be incorporated into the routine follow-up protocol for all MIS-C patients, and those with persistent hypertension should receive appropriate evaluation and management to mitigate long-term cardiovascular risk [60].

Myocardial inhibition and left ventricular systolic dysfunction are common in the acute phase of MIS-C. Most children recover quickly after timely and effective anti-inflammatory treatment. Multiple studies have shown that left ventricular ejection fraction of most children can return to normal within days to weeks [15]. A large cohort study of 1204 MIS-C children found that most children with decreased left ventricular ejection fraction (hospitalization) returned to normal within 6 months [61]. Another mid-term study of 66 MIS-C children reported that all children with left ventricular dysfunction during the acute phase returned to normal within the median 1.5 years following diagnosis [42]. Complete recovery of function does not mean that all children have subclinical abnormalities. Some children may have persistent diastolic dysfunction or exercise endurance decline. For instance, a 6-month follow-up study found that, although the left ventricular systolic function of all the children had normalized, at 2 weeks, 8 weeks, and 6 months, 11%, 9%, and 4% of the children respectively had persistent diastolic dysfunction [55]. Furthermore, more sensitive tools such as cardiac magnetic resonance or strain analysis can reveal subclinical myocardial injury that cannot be detected by conventional echocardiography. Therefore, children with MIS-C show more subclinical myocardial dysfunction during the 6-month follow-up period than COVID-19 children or healthy control groups [41]. Therefore, it is recommended to carry out progressive and supervised cardiac rehabilitation training and regularly assess their exercise capacity by exercise load tests to ensure complete recovery and safe resumption of physical activities. A study has shown that, in MIS-C patients with normal weight, their left ventricular global longitudinal strain (GLS) and ejection fraction (LVEF) continued to show significant improvement during the period from 6 weeks to 6 months after disease onset, with GLS improving from −17.2% at 6 weeks to −19.8% at 6 months and LVEF increasing from 56.3% to 61.5% over the same period, suggesting a continuous process of cardiac functional recovery [62].

### 6.3. Long-Term Multidisciplinary Follow-Up Framework and Patient Education

Establishing a multidisciplinary follow-up team with pediatric cardiologists, rheumatologists, immunologists, and primary care physicians is important for long-term treatment of MIS-C patients [14]. Regular cardiac ultrasound and electrocardiograms are important for monitoring coronary artery disease and cardiac recovery. Monitoring cardiovascular risk factors such as blood lipids and blood pressure is also important to determine whether stress tests or coronary angiography for coronary artery disease are required [57]. A prospective national monitoring study from Switzerland shows that, although left ventricular systolic dysfunction usually resolves quickly, some coronary artery abnormalities persist during follow-up and need longer cardiac tests [63]. Health education for children and families is important for long-term management. Education should cover warning signs such as chest pain and shortness of breath, drug treatment such as aspirin antiplatelet therapy and maintaining a healthy lifestyle such as diet and exercise [64]. Vaccinations such as those for influenza and COVID-19 should also be recommended [65].

## 7. Conclusions

MIS-C is a severe immune-mediated disease caused by SARS-CoV-2 infection and presents clinical challenges and long-term health risks for the heart, including coronary artery damage and myocardial ischemia. Clinically, understanding this spectrum helps us better understand both the post-COVID-19 syndrome in children and immune-mediated vasculitis and myocardial injury.

This review summarizes the current understanding of the disease, encompassing the aberrant immune responses triggered by SARS-CoV-2, the ensuing cytokine storm, vascular endothelial dysfunction, and the associated hypercoagulable state. Similarities and differences between MIS-C and classic Kawasaki disease provide a model for understanding the “common” and “unique” immune response mechanisms to pathogens. There are still gaps in understanding the mechanisms at the level of specific T cells, signaling pathways and genetic susceptibility. Future work should include multiomics technologies for studying the cascade from infection to immune storm and target organ damage at the molecular level, which provides the basis for precise intervention targets.

Diagnosis and treatment are the first step. Echocardiography (heart test) combined with cardiac magnetic resonance imaging (CT) and biomarkers such as NT-proBNP and troponin can be used to detect early risk. Immunomodulatory drugs such as IVIG and glucocorticoids can reduce systemic inflammation, while antiplatelet or anticoagulant agents prevent coronary artery thrombosis and reduce the risk of myocardial ischemia, together improving clinical outcomes. Biochemical drugs like IL-1 and IL-6 receptor antagonists target disease effectively. Optimizing combination, timing and duration depending on disease severity and response will need further clinical studies. On long-term prognosis, most children recover well.

Children with residual coronary artery aneurysms (large ones) are at greater risk of coronary artery stenosis, thrombosis and myocardial ischemia throughout their lives. Lifelong monitoring should be comprehensive and tailored to the severity of coronary artery lesions. Presently, follow-up decisions are often based on guidelines established for Kawasaki disease, but long-term cardiovascular effects of MIS-C may have unique features (for example, persistent effects of myocardial fibrosis and persistent abnormalities in vascular endothelial function) that require long-term prospective cohort studies.

Cardiovascular issues in MIS-C are dealt with both acutely and with a view to long-term risk prevention. Research should balance the fast cognitive development of understanding this disease, i.e., we can use diagnostics and treatments of known diseases like Kawasaki disease, and keep our eyes open to the unique immune and clinical features of MIS-C. Future research should focus on three main areas: immune pathogenesis for new targets, randomized controlled trials for better current treatments and new drugs, and an international collaborative long-term registry for long-term cardiovascular outcomes.

## Figures and Tables

**Table 1:** Comparison of clinical and laboratory features between MIS-C and Kawasaki disease.

Feature	MIS-C	Kawasaki Disease
Age range	Older children and adolescents (median ~8 years)	Infants and young children (median ~2–3 years)
Cardiovascular involvement	Myocardial dysfunction common (50–80%); coronary dilation (15–18%)	Coronary artery aneurysms (15–25%); myocardial dysfunction less common
Shock	Frequent (40–56%); often cardiogenic and distributive	Less common (~5%); Kawasaki disease shock syndrome
Gastrointestinal symptoms	Very common (80–90%); prominent abdominal pain	Less common; milder presentation
Inflammatory markers	Extremely elevated CRP, ferritin; higher IL-6, IL-17A	Elevated CRP; moderate cytokine elevation
Cardiac biomarkers	Markedly elevated troponin and BNP/NT-proBNP	Moderately elevated; lower than MIS-C
Platelet count	Normal or decreased; thrombocytopenia in severe cases	Typically elevated (thrombocytosis) in subacute phase
T-cell activation	Superantigen-mediated broad TRBV11–2 expansion	Conventional antigen-driven; oligoclonal expansion
Coronary outcomes	Usually resolve within weeks to months	May persist for years; risk of stenosis and thrombosis
Response to IVIG	Generally good; may require adjunctive steroids/biologics	Excellent; 10–20% resistant requiring additional therapy

**Table 2:** Imaging modalities for the assessment of coronary artery lesions in MIS-C.

Modality	Primary Indications	Advantages	Limitations
Transthoracic Echocardiography (TTE)	First-line screening and follow-up; coronary diameter measurement (Z-score); ventricular function assessment	Widely available; no radiation; bedside capability; serial monitoring	Limited visualization of mid-distal coronary segments; operator-dependent
Cardiac Magnetic Resonance (CMR)	Myocardial tissue characterization; detection of edema, fibrosis, and perfusion defects; proximal coronary anatomy	Excellent tissue resolution; no ionizing radiation; quantitative T1/T2 mapping; functional assessment	Limited availability; long acquisition time; may require sedation in young children
Coronary CT Angiography (CCTA)	Detailed coronary artery anatomy; mid-distal segment evaluation; calcification detection	High spatial resolution; rapid acquisition; excellent for distal coronary visualization	Ionizing radiation exposure; requires breath-hold; contrast nephrotoxicity risk
Stress Echocardiography/CMR	Evaluation of inducible myocardial ischemia; functional reserve assessment	Physiological assessment of coronary flow reserve; detects subclinical ischemia	Limited pediatric data; technically challenging

**Table 3:** Summary of acute treatment strategies for MIS-C.

Therapy	Agent(s)	Indication	Key Considerations
Immunomodulation (first-line)	IVIG (2 g/kg); Methylprednisolone (2–10 mg/kg/day)	All confirmed MIS-C cases	Combined IVIG + steroids may provide more rapid resolution than IVIG alone
Antiplatelet therapy	Low-dose aspirin (3–5 mg/kg/day, max 100 mg)	All MIS-C patients during acute and subacute phases	Continue until coronary arteries normal; avoid in active bleeding or severe thrombocytopenia
Anticoagulation	Enoxaparin (1 mg/kg BID); Warfarin (target INR 2.0–3.0)	Moderate to large coronary artery aneurysms (Z-score >= 5.0); documented thrombosis	Requires monitoring of anti-Xa levels or INR; consider bridging with UFH in acute setting
IL-1 receptor antagonist	Anakinra (2–10 mg/kg/day SC/IV)	Refractory cases; severe hyperinflammation; persistent shock	Rapid onset of action; monitor for infection; limited pediatric data
IL-6 receptor antagonist	Tocilizumab (8–12 mg/kg IV)	Refractory cases with prominent IL-6 elevation; steroid-resistant disease	Single-dose regimen often effective; risk of secondary infections; transient transaminitis
Vasoactive support	Milrinone; Epinephrine; Norepinephrine; Dobutamine	Cardiogenic shock; distributive shock; myocardial dysfunction	Guided by hemodynamic monitoring; consider ECMO in refractory shock

## Data Availability

No new data were created or analyzed in this study. Data sharing is not applicable to this article.

## Abbreviations

The following abbreviations are used in this manuscript:

MIS-C	Multisystem inflammatory syndrome in children
CMR	Cardiac magnetic resonance imaging
CCTA	Coronary CT angiography
